# Positive anti-citrullinated protein antibody status and small joint arthritis are consistent predictors of chronic disease in patients with very early arthritis: results from the NOR-VEAC cohort

**DOI:** 10.1186/ar2820

**Published:** 2009-10-01

**Authors:** Maria D Mjaavatten, Till Uhlig, Anne J Haugen, Halvor Nygaard, Göran Sidenvall, Knut Helgetveit, Tore K Kvien

**Affiliations:** 1 Department of Rheumatology, Diakonhjemmet Hospital, P.O. box 23 Vinderen, 0319 Oslo, Norway; 2 Department of Rheumatology, Østfold Hospital Trust, 1603 Fredrikstad, Norway; 3 Lillehammer Hospital for Rheumatic Diseases, Margrethe Grundtvigs vei 6, 2609 Lillehammer, Norway; 4 Department of Rheumatology, Innlandet Hospital, 2226 Kongsvinger, Norway; 5 Martina Hansen Hospital, P.O. box 23, 1306 Bærum Postal Terminal, Norway

## Abstract

**Introduction:**

The current 1987 American College of Rheumatology (ACR) classification criteria for rheumatoid arthritis (RA) have proven less useful in early arthritis. The objective of this study was to identify and compare predictors of three relevant outcomes of chronic arthritis in a cohort of very early arthritis patients.

**Methods:**

The Norwegian Very Early Arthritis Cohort (NOR-VEAC) includes adult patients with at least one swollen joint of ≤16 weeks' duration. Patients are followed for 2 years with comprehensive clinical and laboratory examinations. Logistic regression analyses were performed to determine independent predictors of three outcomes: persistent synovitis, prescription of disease-modifying anti-rheumatic drugs (DMARDs), and established clinical RA diagnosis within one year.

**Results:**

Of 384 patients eligible for one year follow-up (56.3% females, mean (SD) age 45.8 (14.7) years, median (IQR) duration of arthritis 31 (10-62) days), 14.4% were anti-CCP2 positive, and 11.2% were IgM RF positive. 98 patients (25.5%) had persistent synovitis, 106 (27.6%) had received DMARD treatment during follow-up, while 68 (17.7%) were diagnosed with RA. Consistent independent predictors across all three outcomes were positive anti-citrullinated protein antibody (ACPA) status (odds ratio (OR) 3.2, 5.6 and 19.3), respectively, and small joint arthritis (proximal interphalangeal joint (PIP), metacarpo-phalangeal joint (MCP), and/or metatarso-phalangeal joint (MTP) joint swelling) (OR 1.9, 3.5, and 3.5, respectively).

**Conclusions:**

Positive ACPA status and small joint arthritis were consistent predictors of three relevant outcomes of chronic arthritis in very early arthritis patients. This consistency supports DMARD prescription as a valid surrogate endpoint for chronic arthritis. Importantly, this surrogate is used in ongoing efforts to develop new diagnostic criteria for early RA.

## Introduction

The 1987 American College of Rheumatology (ACR) classification criteria for rheumatoid arthritis (RA) were designed to ensure that patients included in clinical trials were true RA patients, and the criteria have been of major importance to clinical research in rheumatology [1]. However, the criteria were never intended to be used for RA diagnosis, and many studies have shown that the existing criteria lack sensitivity and are more useful in established rather than early arthritis [2-5]. Nevertheless, the criteria have been widely used in the clinical setting, even in the assessment of patients with recent-onset arthritis, and it can be assumed that rheumatologists often consider the fulfilment of these criteria when evaluating the diagnosis and/or prognosis in their arthritis patients [6]. Features such as erosions and rheumatoid nodules reflect established disease and their value as part of the criteria is questioned in the era of early aggressive treatment, when the aim is to treat patients before bone damage occurs.

Several authors have called for new classification criteria [7-9]. The target patient group in which potential new criteria will be applied is probably wider and more diverse than patients fulfilling the current ACR criteria alone. Persistent synovitis as a marker of chronic disease is also an outcome of interest in early inflammatory arthritis [10-12], although RA development in itself is important to predict. An ongoing European League Against Rheumatism (EULAR)/ACR task force aims to define a set of criteria for the diagnosis of early RA [13]. To avoid circularity, the task force has proposed to use the start of DMARD therapy as a surrogate endpoint in the data-driven process of developing new candidate criteria. The rationale behind this approach is derived from the hypothesis that the physician at the time of a disease-modifying anti-rheumatic drug (DMARD) prescription assumes the patient to be at high risk of developing chronic and severe disease.

The purpose of this study was to determine predictors of three relevant outcomes in early arthritis: persistent joint swelling, DMARD prescription, and RA development.

## Materials and methods

### Early arthritis clinic

The Norwegian Very Early Arthritis Clinic (NOR-VEAC) study was started in 2004 as a multicenter observational study in the South-Eastern part of Norway. The five participating hospitals serve a region with approximately 1.7 million inhabitants. The cohort includes patients (age 18 to 75 years) presenting with at least one clinically swollen joint of 16 weeks' duration or less, and patients are followed longitudinally for two years.

Patients with joint swelling due to trauma, osteoarthritis, crystal arthropathies, and septic arthritis are excluded from follow-up. The study was approved by the regional Ethics Board and the Data Inspectorate, and patients gave informed consent.

### Data collection

Data collection was performed by rheumatologists and designated study nurses in the five different centers. Registration included age, sex, duration of symptoms, co-morbidities, extra-articular symptoms, level of education, occupational status, smoking and coffee drinking habits, height and weight. Sixty-eight swollen joint counts (SJC) and 28 tender joint counts (TJC) were performed by a rheumatologist or by experienced study nurses. Patient-reported outcomes included joint pain, fatigue, and global health status on visual analogue scales (VAS), the Norwegian versions of the Health Assessment Questionnaire (HAQ) [14], and Short Form Health Survey (SF-36) with aggregated physical and mental component summary scores [15,16]. Information about morning stiffness (duration) was captured from the Rheumatoid Arthritis Disease Activity Index (RADAI) [17]. The assessor reported global evaluation of disease activity on a VAS. Erythrocyte sedimentation rate (ESR) and C-reactive protein (CRP) levels were determined at the local laboratories. Serum was frozen and stored at -70°C and analysed in one batch for anti-citrullinated protein antibodies (ACPA; anti-CCP2^®^, INOVA Diagnostics, Inc. San Diego, CA, USA) and IgM rheumatoid factor (RF; using ELISA). The cut-off levels for positivity of serologic markers recommended by the central laboratory were employed, as also reported previously (anti-CCP2 ≥25 units/ml, IgM RF ≥25 IU/ml) [18].

### Outcome variables

Patients eligible for one year of follow-up time by 1 January, 2008, were included in the current analyses. Persistent synovitis was defined as presence of one or more swollen joints on at least two out of three follow-up assessments during the first year. Synovitis did not have to be present in the same joint on consecutive assessments. Patients lost to follow-up with less than two recorded follow-up visits were assumed to have non-persistent arthritis. A clinical diagnosis of RA was recorded by the treating rheumatologist, and diagnosis was therefore not restricted to fulfilment of the ACR classification criteria. Information about DMARD prescription was collected through chart review, and start of DMARD(s) during the first year and DMARD type were recorded. Patients lost to follow-up were assumed to neither have RA nor have received DMARD treatment after the last recorded visit.

### Statistical analysis

Means and standard deviations were calculated for continuous variables following a Gaussian distribution, otherwise median values and interquartile ranges (25th to 75th percentiles) were calculated. Multiple logistic regression analyses were used to determine baseline variables independently associated with each outcome (dependent variables). The independent variables were selected from univariate analyses if *P *< 0.25. The model building process was performed according to the methods described by Hosmer and Lemeshow [19]. Sex, ACPA status, RF status, morning stiffness lasting less than one hour, presence of joint swelling in either metacarpo-phalangeal joint (MCP), proximal interphalangeal joint (PIP), or metatarso-phalangeal joint joint (MTP) joint(s) (small joint arthritis), and smoking status (never/ever) were entered as dichotomous variables. The following variables were entered as continuous measures in the regression analyses: age, 68-SJC, 28-TJC, ESR, CRP, HAQ. All tests were conducted at the 0.05 significance level. Analyses were performed using SPSS 14.0 (SPSS, Chicago, IL, USA).

## Results

### Demographic, clinical and biological characteristics

Baseline characteristics of the 384 patients with early arthritis included in the present analyses are reported in Table 1. The duration of joint swelling at inclusion was very short (median 31 days), and 14.3% were ACPA positive and 11.4% were IgM positive. Percentages of ACPA/IgM RF positive patients in each of the groups according to outcome were: persistent arthritis 35.2/26.7, DMARD prescription 41.5/31.5, and RA 58.8/49.2. Corresponding percentages of patients with elevated ESR/CRP in the different outcome groups were 59.8/55.1, 67.9/59.4, and 61.8/51.5, respectively. Monoarthritis at presentation was seen in 146 (38%) patients, 130 (33.9%) patients had two to four swollen joints (oligoarthritis), and 108 (28.1%) patients presented with polyarthritis. Twenty-four (16%) patients in the persistent arthritis group, 5 (3%) patients in the RA group and 17 (16%) patients in the DMARD prescription group presented with monoarthritis. The knee joint was the most frequently involved single joint (39.1%), followed by the ankle (32.3%) and wrist (30.2%) joints. Small joints in hands and feet were involved in 43.8% of patients.

**Table 1:** Baseline characteristics of 384 patients with early arthritis

	Mean (SD)/n (%)	Median (IQR)
Female gender	217 (56.5)	
Age (years)	45.8 (14.7)	44.8 (34.7-58.1)
Arthritis duration (days)	38.0 (30.0)	31 (10-62)
SJC (0-68)	4.5 (6.7)	2.0 (1-5)
TJC (0-28)	3.2 (5.0)	1.0 (0-4)
ESR (mm/h)	31 (24)	24 (12-48)
ESR >20 mm/h	214 (56.0)	
CRP (mg/l)	32 (47)	15 (5-43)
CRP >10 mg/l	224 (58.3)	
IgM RF positive	38 (11.4)	
ACPA positive	49 (14.3)	
Assessor's global VAS (mm)	36 (21)	32 (19-50)
Patient's global VAS (mm)	52 (25)	53 (35-71)
Joint pain VAS (mm)	51 (26)	52 (29-72)
Fatigue VAS (mm)	40 (29)	40 (11-65)
Morning stiffness >1 hour	195 (50.8)	
DAS28	4.01 (1.34)	3.90 (3.06-4.82)
HAQ (0-3)	0.84 (0.67)	0.75 (0.25-1.25)
SF-36:		
PCS	33.4 (10.8)	32.8 (25.1-40.4)
MCS	48.6 (11.4)	50.4 (40.1-57.4)
Ever smoker	236 (61.5)	

Number of patients with missing data: SJC/TJC 0, CRP 0, ESR 2, HAQ 9, CCP 42, IgM RF 51, smoking 3, morning stiffness 9, small joints 0.

ACPA = anti-citrullinated protein antibody; CRP = C-reactive protein; DAS = Disease Activity Score; ESR = erythrocyte sedimentation rate; HAQ = Health Assessment Questionnaire; IQR = inter-quartile range; MCS = Mental Component Summary; PCS = Physical Component Summary; RF = rheumatoid factor; SD = standard deviation; SF-36 = Short Form Health Survey; SJC = swollen joint count; TJC = tender joint count; VAS = visual analogue scale.

Of 384 included patients, 287 (75%) completed the one year follow-up. Recorded reasons for drop-out (number of patients) were remission (43), lost contact with patient (24), other diagnoses (6), patient moved (2), other (2), and unknown (20).

### Frequency of outcomes

**Figure 1:**
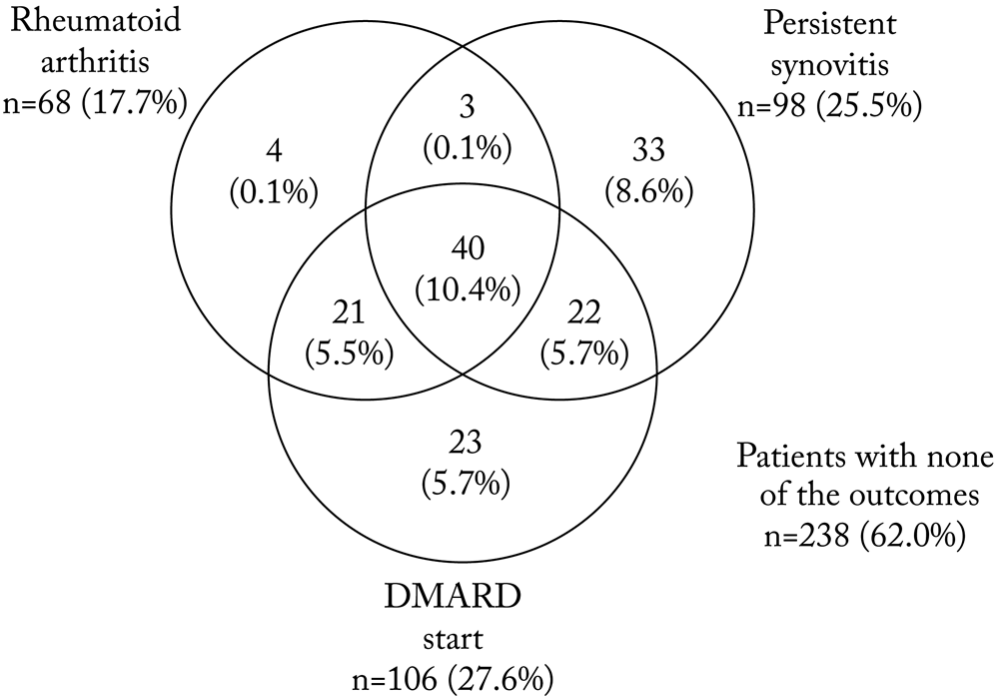
Persistent synovitis, DMARD prescription, and rheumatoid arthritis diagnosis as one-year outcomes in 384 patients with very early arthritis. DMARD = disease-modifying anti-rheumatic drugs.

A total of 146 patients had one or more of the assessed outcomes at one year. The distribution of patients by outcome is depicted in Figure 1. Forty patients (10.4%) had all three outcomes. Only seven patients with RA had not been prescribed DMARD treatment during the first year, compared with 33 patients with persistent synovitis, whereas 45 (12%) patients were prescribed DMARD therapy without a RA diagnosis. Although 68 (17.7%) patients had a RA diagnosis at one year, an additional 78 (20.3%) patients had either persistent synovitis or had received DMARD therapy, and thus had a chronic inflammatory joint condition without being diagnosed with RA. None of the outcomes were seen in 238 patients. The distribution of diagnoses during follow-up in these patients were (last observation carried forward): undifferentiated arthritis 118 (49.6%), reactive arthritis 52 (21.8%), sarcoidosis-related arthritis/Löfgren's syndrome 44 (18.5%), psoriatic arthritis 11 (4.6%), osteoarthritis 5 (2.1%), gout 2 (0.8%), and other 6 (2.5%).

### Persistent synovitis

Ninety-eight (25.5%) patients had persistent synovitis during follow-up. Joint counts, positive ACPA or RF status, morning stiffness, HAQ, and higher age were factors associated with persistent synovitis in the univariate analyses (Table 2). Independent predictors of persistent synovitis were positive ACPA status, involvement of small joints in hands and/or feet and HAQ. A higher CRP at presentation was unexpectedly protective of having persistent synovitis throughout the first year (*P *= 0.05; Table 3).

**Table 2:** Prediction of persistent synovitis, DMARD prescription and RA at one year in univariate logistic regression analyses

	Dependent variable					
	
	Persistent synovitis n = 98		DMARD prescription n = 106		RA n = 68	
	
Baseline variables	OR (95% CI)	*P *value	OR (95% CI)	*P *value	OR (95% CI)	*P *value
Age (years)	1.02 (1.01-1.04)	0.008*	1.02 (1.00-1.04)	0.017*	1.05 (1.03-1.07)	< 0.001*
Female gender	1.46 (0.91-2.33)	0.12*	1.06 (0.67-1.67)	0.80	1.12 (0.67-1.91)	0.67
68-SJC	1.08 (1.03-1.12)	< 0.001*	1.14 (1.09-1.20)	< 0.001*	1.15 (1.09-1.21)	< 0.001*
28-TJC	1.11 (1.06-1.16)	< 0.001*	1.19 (1.13-1.26)	< 0.001*	1.20 (1.13-1.27)	< 0.001*
ESR (mm/h)	1.00 (1.00-1.01)	0.41	1.01 (1.00-1.02)	0.09*	1.00 (0.99-1.01)	0.98
CRP (mg/l)	1.00 (0.99-1.00)	0.18*	1.00 (1.00-1.01)	0.80	1.00 (0.99-1.00)	0.23*
ACPA positivity	7.13 (3.73-13.64)	< 0.001*	16.9 (7.94-35.8)	< 0.001*	57.6 (24.6-134.5)	< 0.001*
IgM RF positivity	5.65 (2.78-11.5)	< 0.001*	11.8 (5.34-26.4)	< 0.001*	28.5 (12.3-65.8)	< 0.001*
Morn. stiffness >1 hour	1.74 (1.08-2.79)	0.023*	2.71 (1.68-4.38)	< 0.001*	2.47 (1.40-4.37)	0.002*
HAQ (0-3)	1.61 (1.15-2.27)	0.006*	2.22 (1.57-3.14)	< 0.001*	1.63 (1.11-2.39)	0.012*
Small joint arthritis^§^	3.72 (2.29-6.05)	< 0.001*	8.19 (4.83-13.9)	< 0.001*	20.5 (8.58-48.9)	< 0.001*
Ever smoker	1.19 (0.74-1.92)	0.48	1.33 (0.83-2.13)	0.24*	2.71 (1.44-5.09)	0.002*

^§ ^Metacarpo-phalangeal joint, proximal interphalangeal joint, or metatarso-phalangeal joint joint swelling.*P < 0.25, variable selected for inclusion in multivariate analyses.

ACPA = anti-citrullinated protein antibody; CI = confidence interval; CRP = C-reactive protein; DMARD = disease-modifying anti-rheumatic drugs; ESR = erythrocyte sedimentation rate; HAQ = Health Assessment Questionnaire; OR = odds ratio; RA = rheumatoid arthritis; RF = rheumatoid factor; SJC = swollen joint count; TJC = tender joint count.

**Table 3:** Prediction of persistent synovitis, DMARD prescription and RA in final multivariate logistic regression models

	Dependent variable					
	
	Persistent synovitis n = 98		DMARD start n = 106		RA n = 68	
	
Baseline variables	OR (95% CI)	*P *value	OR (95% CI)	*P *value	OR (95% CI)	*P *value
Age (years)	1.01 (0.99-1.03)	0.18	1.00 (0.98-1.02)	0.70	1.04 (1.01-1.08)	0.018
Female gender	1.20 (0.68-2.12)	0.52	0.99 (0.54-1.79)	0.97	1.24 (0.51-3.01)	0.64
ACPA positivity	4.50 (2.17-9.33)	< 0.001	8.11 (3.55-18.6)	< 0.001	19.3 (6.84-54.4)	< 0.001
IgM RF positivity	-	-	-	-	5.02 (1.47-17.1)	0.010
Small joint arthritis^§^	2.11 (1.17-3.81)	0.013	3.86 (2.02-7.37)	< 0.001	3.45 (1.21-9.90)	0.021
HAQ	1.73 (1.12-2.68)	0.014	1.75 (1.13-2.74)	0.012	-	-
28-TJC	-	-	1.06 (1.00-1.13)	0.064	1.09 (1.02-1.16)	0.012
CRP (mg/l)	0.99 (0.98-1.00)	0.042	-	-	-	-
Constant	0.069	-	0.075	-	0.003	-

^§ ^Metacarpo-phalangeal joint, proximal interphalangeal joint, or metatarso-phalangeal joint joint swelling.

ACPA = anti-citrullinated protein antibody; CI = confidence interval; CRP = C-reactive protein; DMARD = disease-modifying anti-rheumatic drugs; HAQ = Health Assessment Questionnaire; OR = odds ratio; RA = rheumatoid arthritis; RF = rheumatoid factor; TJC = tender joint count.

### DMARD prescription

Treatment with DMARDs was initiated during the first year in 106 (27.6%) patients. Methotrexate was the predominant drug of choice, and was given as monotherapy in 64 (16.7%) patients. Biological agents were given to 13 patients, all in combination with methotrexate. Sulphasalazine was prescribed in 10 (2.6%) patients, leflunomide monotherapy in 1 patient, and combinations of traditional DMARDs were given in 18 (4.7%) patients. Joint counts, ACPA, RF, morning stiffness, HAQ, and small joint arthritis were univariately associated with initiation of DMARD therapy during follow-up (Table 2). Multivariate regression analysis showed that ACPA positivity, small joint arthritis, and HAQ were independent predictors of DMARD prescription. Tender joint count also contributed to the final model shown in Table 3.

### Rheumatoid arthritis

Of the 68 (17.7%) patients who were diagnosed with RA within the first year of follow-up, 36 were diagnosed at the first visit. Univariate associations were significant for the same variables for RA as for persistent synovitis, with the addition of smoking (Table 2). The multivariate regression model (Table 3) showed that age, ACPA, RF, tender joint count and small joint involvement were independently associated with a RA diagnosis within the first year of follow-up. Evidence of confounding between the serologic markers was found as removal of RF resulted in an increase in the regression coefficient of ACPA exceeding 15%.

A general perception is that patients with monoarthritis rarely develop RA. Our results regarding predictors for RA were maintained also in a reanalyses of the cohort after excluding the patients with monoarthritis at baseline (data not shown).

## Discussion

Identification of patients with recent-onset arthritis at high risk of developing chronic arthritis is an important clinical challenge, because individual management of early arthritis should be based on the expected disease course. One particular difficulty is to decide the most relevant outcome when prediction models are developed in undifferentiated patient populations. RA development is probably not the only important endpoint, as some patients with undifferentiated, persistent arthritis have been shown to have a prognosis similar to RA patients [20]. The clinical utility of prediction models is presumably enhanced if predictors are similar across several relevant endpoints. In the present study, we found that ACPA and arthritis of joints in fingers or toes were consistent predictors regardless of whether the outcome was persistent synovitis, start of DMARD treatment, or RA diagnosis.

Some previous studies have identified ACPA as a significant predictor of transition from undifferentiated arthritis to RA [21-23]. A study in undifferentiated arthritis conducted without measuring ACPA found RF to be predictive of RA [24]. Van der Helm-van Mil and colleagues found individual contribution of both RF and anti-CCP to RA development in their study [22]. This is in line with our findings, where both RF and ACPA were independently associated with RA diagnosis. For RA, both RF and anti-CCP have been found to be present in premorbid sera several years before disease start, and these biomarkers are therefore especially important in very early arthritis [25-27]. Results of the PROMPT study even suggested that ACPA-positive early arthritis is a distinct disease entity whose prognosis can be altered by early treatment with methotrexate [28].

Arthritis of the hand joints is part of the original classification criteria for RA [1]. Emery and colleagues recognised arthritis of the MTP joints, diagnosed by the so-called squeeze test, as a clinical sign requiring early referral to a rheumatologist for evaluation of possible RA [29]. This led us to investigate the separate predictive capacity of the involvement of small joints inhand and feet joints. We found that small joint involvement of hands and feet were separately weaker predictors across all three outcomes than hands and feet combined (data not shown).

Prescription of DMARD therapy in early arthritis has also been previously used as a surrogate for chronic arthritis, but Quinn and colleagues were unable to find baseline predictors for this endpoint [24]. According to the 'window-of-opportunity' hypothesis, DMARD treatment should ideally be initiated within three months of symptom onset in high-risk patients [30]. It is therefore crucial to define predictive characteristics that are present at a very early stage of disease. The NOR-VEAC cohort has median arthritis duration of only 31 days, and this short disease duration, together with the wide inclusion criteria and a comprehensive data collection, offers a unique opportunity for prediction analysis. However, radiographic data were not available for these patients, and this represents a limitation of our study. Erosive disease has been shown to occur early and to predict long-term disability [31,32]. Prediction of erosive disease is therefore essential, because DMARD treatment can ameliorate prognosis in high-risk patients [33-35]. Future follow-up results of the NOR-VEAC cohort will supplement current knowledge in this field, as imaging procedures were included in the data collection in 2007.

As data on erosions were not available, fulfilment of the ACR criteria for RA could not be formally assessed in this study. Although more subjective, a clinical diagnosis of RA based on the judgment of an experienced rheumatologist has been shown to be reliable [36], and use of this outcome can certainly be defended from a clinical point of view.

ACR and EULAR are now developing new classification criteria for early RA. The data-driven part of this process includes longitudinal data from several early arthritis cohorts, and is conducted with DMARD start as a surrogate endpoint for chronic disease. Our results confirm that RA patients and patients prescribed with DMARDs have common characteristics, and this supports the validity of the strategy. This is further supported by the fact that in our study patients taking DMARDs were a larger group than RA patients alone: as many as 45 patients (11.4%) in our material were prescribed DMARDs without having received an RA diagnosis (Figure 1). These patients probably deserve to be recognised by new criteria. The ACR/EULAR task force has decided to only focus on the subset of patients started on methotrexate. Methotrexate was the predominant DMARD prescribed in our patients, and analyses with methotrexate start as the dependent variable yielded similar results with regard to predictors (data not shown).

Persistent synovitis as an outcome in early arthritis can be defined in different ways. Harrison and Symmons studied different outcomes in inflammatory arthritis in the Norfolk Arthritis Register and defined remission of synovitis as no soft tissue joint swelling and no treatment with DMARDs or steroids within the previous three months [10]. RF, tender joint count more than 6, and presence of ankle synovitis were independent predictors of persistent synovitis. Visser and colleagues used a similar definition of 'natural remission' in the Leiden Early Arthritis Clinic [11]. We chose to define persistent synovitis and DMARD initiation as two separate outcomes. This approach allowed 23 patients without joint swelling at one year, but who were on DMARD therapy, to be excluded from the persistent synovitis group (Figure 1).

However, the independent predictors were similar for both outcomes which suggests that the difference in our definition of persistent disease from that used in previous studies had little impact on the results with regard to predictors. A higher CRP at presentation was found to be protective of persistent synovitis in this study. Although this is contrary to what is generally reported in the literature, Green and colleagues in their study of 63 early arthritis patients also found a protective effect of elevated CRP for persistent arthritis [37]. They suggested that a sub-entity of patients with 'an acute-onset, polyarticular disease, associated with an elevated CRP, that responds to corticosteroids in the early stages' may exist. Another possible explanation for the protective effect of CRP is that patients presenting with monoarthritis of the large joints tend to have high CRP levels, but are less prone to develop persistent arthritic disease.

When assessing predictors in early arthritis populations, the issue of circularity of reasoning is relevant. One can argue that presence of ACPA and small joint involvement will indeed influence both the clinician's diagnosis of RA and the decision to prescribe a DMARD. However, in our cohort, analysis of anti-CCP was performed as a batch in frozen sera, so knowledge of this biomarker was not routinely available to the clinicians at the time of clinical decision-making, although the clinicians were free to request this analysis as a part of the work-up. Moreover, the same predictors were also found to be predictive of an outcome independent of the clinician's knowledge of these factors, namely persistent joint swelling. We believe that the use of this additional outcome in our study adds to the validity of the results.

## Conclusions

A few studies have investigated predictors of several different outcomes in early arthritis, but as far as we know, this study is the first to identify consistent predictors of both RA, DMARD prescription, and persistent synovitis. In conclusion, our results show that ACPA positive early arthritis with involvement of small joints in the extremities is likely to progress to persistent disease. As well as supporting the approach chosen by the ACR/EULAR task force in defining new criteria, our study emphasises the need for new criteria for early inflammatory arthritis. Sixty-eight patients in our material were diagnosed with RA during one year of follow-up. More importantly, an additional 78 patients (20%) either received DMARD therapy or had persistent joint swelling on repeated assessments without receiving a RA diagnosis. These patients should probably be recognised within the framework of new criteria.

## Abbreviations

ACPA: anti-citrullinated protein antibody; ACR: American College of Rheumatology; CCP: cyclic citrullinated peptide; CRP: C-reactive protein; DMARDs: disease-modifying anti-rheumatic drugs; ELISA: enzyme-linked immunosorbent assay; ESR: erythrocyte sedimentation rate; EULAR: European League Against Rheumatism; HAQ: Health Assessment Questionnaire; MCP: metacarpo-phalangeal joint; MTP: metatarso-phalangeal joint; NOR-VEAC: Norwegian Very Early Arthritis Cohort; PIP: proximal interphalangeal joint; RA: rheumatoid arthritis; RADAI: Rheumatoid Arthritis Disease Activity Index; RF: rheumatoid factor; SF-36: Short Form Health Survey; SJC: small joint count; TJC: tender joint count; VAS: visual analogue scale.

## Competing interests

The authors declare that they have no competing interests.

## Authors' contributions

MDM performed the statistical analyses and drafted the manuscript, as well as participating in the study design. TU helped to draft the manuscript and participated in the data collection. AJH, HN, and KH participated in the study design and data collection. GS participated in the data collection. TKK was the main designer of the study and helped draft the manuscript. All authors read and approved the final manuscript.
